# Intradural abscess: A challenging diagnosis. Case series and review of the literature

**DOI:** 10.1016/j.radcr.2023.08.084

**Published:** 2023-09-13

**Authors:** Allegra Romano, Antonella Blandino, Andrea Romano, Serena Palizzi, Giulia Moltoni, Michele Acqui, Massimo Miscusi, Alessandro Bozzao

**Affiliations:** a Neuroradiology Unit, Sant'Andrea Hospital, Department of Neuroscience, Mental Health and Sense Organs (NESMOS), Sapienza University of Rome, Rome, Italy; b Division of Neurosurgery, Sant'Andrea Hospital, Department of Neuroscience, Mental Health and Sense Organs (NESMOS), Sapienza University of Rome, Rome, Italy

**Keywords:** Magnetic resonance imaging, Intradural abscess, Lumbar spine

## Abstract

Spinal intradural abscesses are extremely rare. To our knowledge, only a few cases have been described in the literature. We report 2 cases of spinal intradural abscesses in patients presenting to our institution with different symptomatology. Both cases involved the lumbar spine, with different etiologies: Case 1 was presumptively related to spondylitis phenomena, with surgery confirming the intradural localization of the abscess; case 2 was of probable iatrogenic nature (secondary to lumbar drain positioning). The aim of this report is to briefly discuss the clinical significance and pathogenesis of these cases.

## Introduction

Spinal intradural abscesses are an extremely rare condition. To our knowledge, only a few cases have been described in the literature with long-term sequelae (eg, neurological impairment) and poor prognosis if untreated [1]. Most commonly, intradural abscesses have been described as a consequence of a hematogenous spread of an infection, however, they may also be secondary to iatrogenic causes (eg, epidural injections) or to spondylitis phenomena [2].

We would like to report 2 cases of patients presenting to our institution with different symptomatology and a common diagnosis of intradural spinal collections.

## Case 1

### Medical history and examination

A 71-year-old female patient presented to our attention with a 1-month history of progressively disabling and intense back pain with bilateral cruralgia, not responsive to medical therapy. Neurological examination revealed chronic lateral hypoesthesia of the right thigh. Blood tests showed elevated values of C-reactive protein, normal white blood cell count with mild relative neutrophilia, and relative lymphopenia.

**Fig. 1:**
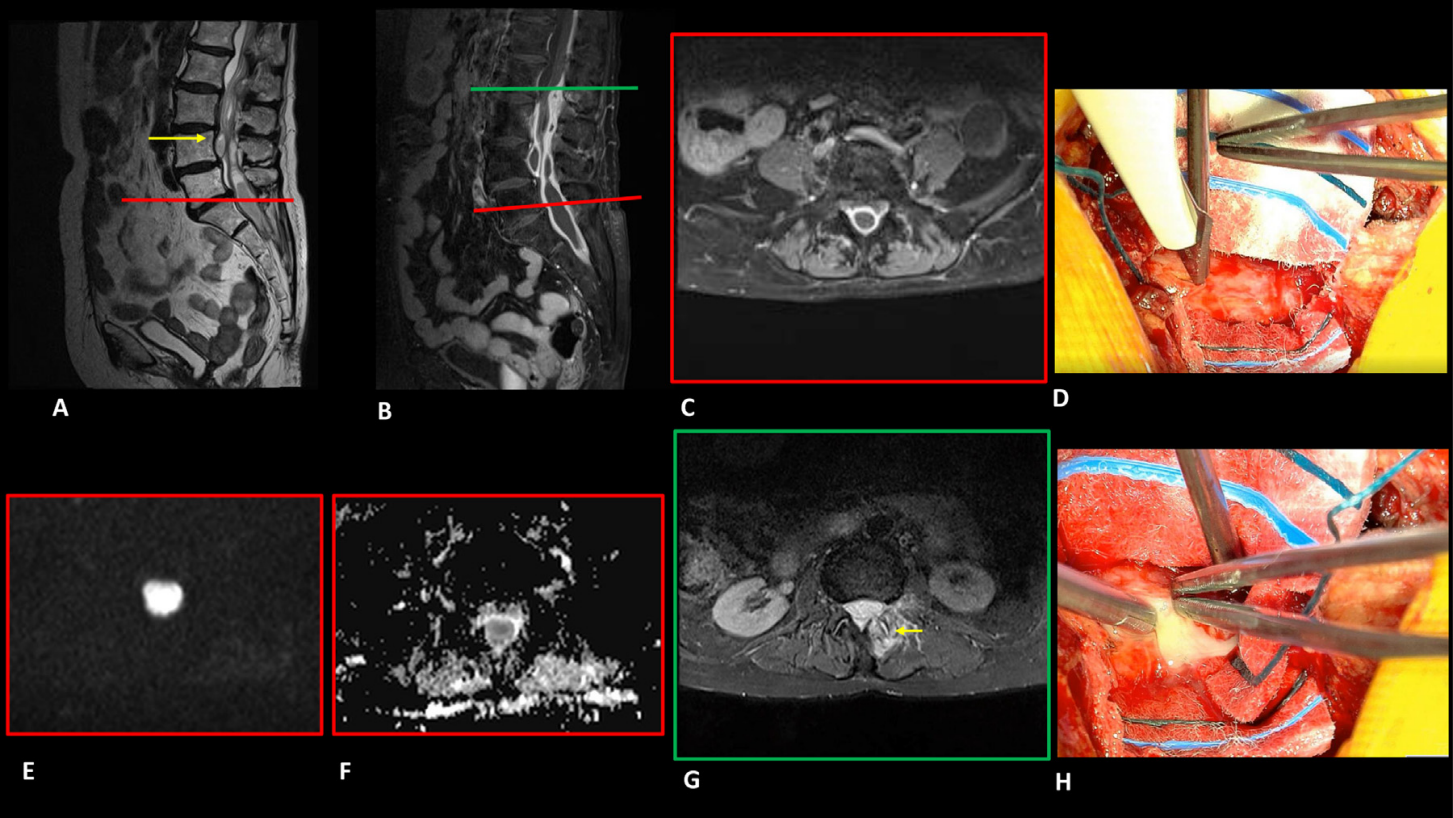
Magnetic resonance imaging of the lumbar spine. Sagittal T2 weighted MRI image (A) reveals 2 suspected intradural collections localized ventrally and dorsally to caudal roots, with hypointense margins and an internal inhomogeneous hyperintense component. Postcontrast sagittal (B) and axial (C) T1 weighted image showing intense peripheral postcontrast enhancement and internal colliquative appearance of the suspected intradural collections at L2-S2 level. Axial DWI(E)/ADC(F) demonstrating restriction within the mass. Axial (G) STIR sequence revealing pathological hypersignal of the left facet joint at level L2-L3 extended to the lamina and spinous process of L2 as well as to the musculature of the contiguous paravertebral lodge. Intraoperative photographs (D–H), (D) A L5-S1 laminectomy was performed. The epidural space and the dural surface appeared undamaged, proving the absence of epidural collections. A vertical durotomy was performed, which showed an intact fibrous capsule. (H) Yellowish purulent material oozing out after incision of the fibrous capsule, confirming the intradural location of the abscess.

MRI of the spine demonstrated the presence of suspected intradural collections in the lumbosacral region at the L2-S2 level with low values of ADC, intense peripheral enhancement after gadolinium injection, and internal colliquative appearance. There was a pathological dural enhancement extended cranially up to T9-T10. In addition, there was a focal inflammatory/infective spondylitis involving the left facet joint at the level L2-L3 (Fig. 1).

During surgery, a durotomy was therefore necessary, confirming the radiological suspicion of the intradural location of the collections.

### Pathological findings and postoperative course

Postoperatively, the patient received empirical intravenous antibiotic treatment with vancomycin, ceftriaxone, and metronidazole, awaiting cultures. Follow-up MRI performed 2 days after the surgery showed markedly decreased abscess size.

The biological examination of the purulent sample did not show any micro-organism growth neither blood cultures were positive.

The last follow-up MRI (2 months later) showed the disappearance of lumbar intradural collections, with the presence of arachnoiditis as a sequela.

## Case 2

**Fig. 2:**
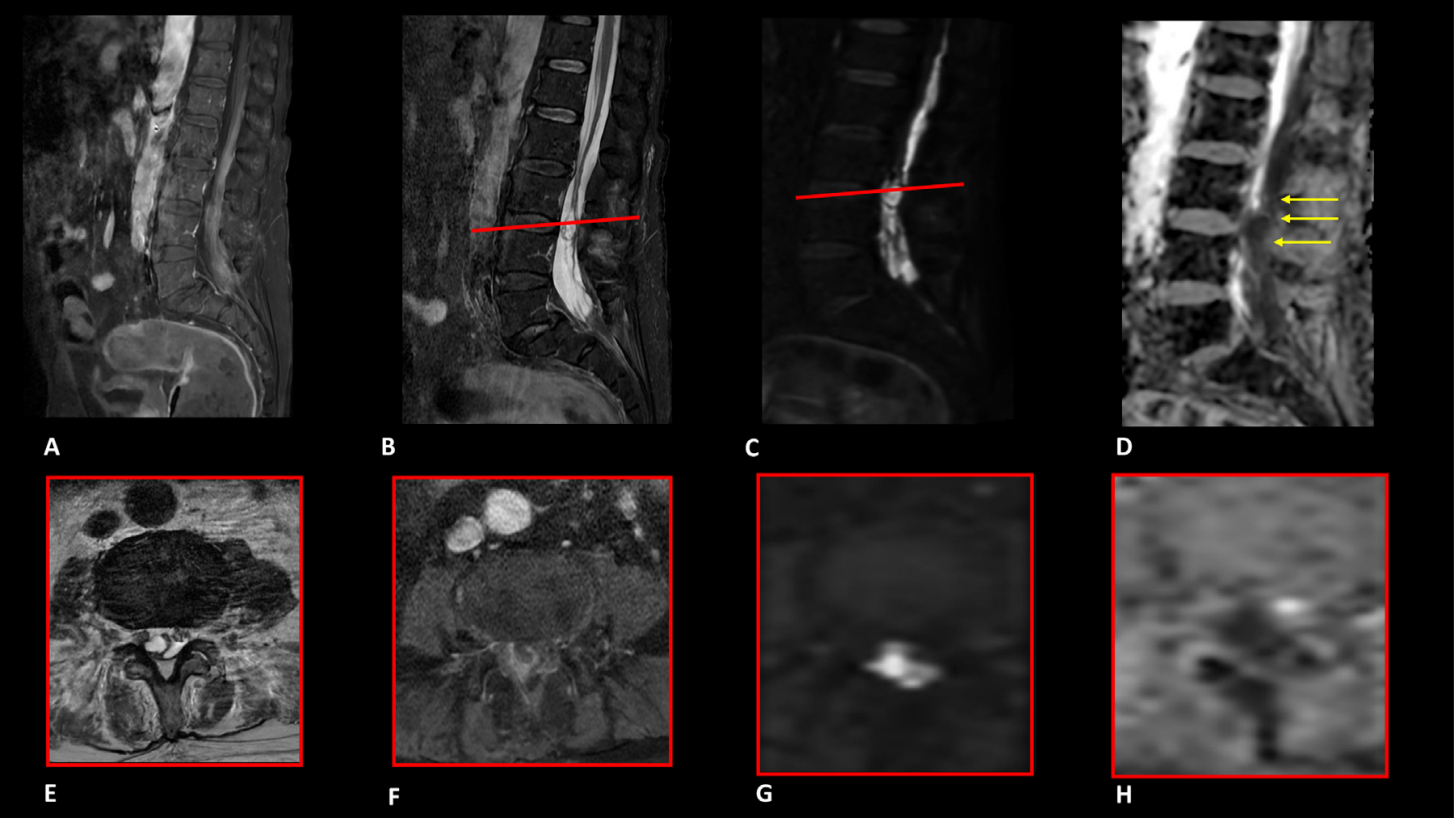
Magnetic resonance imaging of the lumbar spine. Postcontrast sagittal (A) and axial (F) T1 weighted image showing mild post-contrastographic enhancement of a suspected intradural collection in the lumbosacral region (L3-L4 level); Sagittal (C) and axial (G) DWI showed marked restricted diffusion with marked hyposignal on ADC (sagittal D and axial H); in addition, there were signs of arachnoiditis affecting the roots of the cauda which were particularly glued together, in an antigravity position, best appreciated on sagittal STIR (B) and T2 axial image (E).

A 70-year-old male patient with a history of a kidney tumor and brain meningiomatosis was treated with surgeries and stereotactic radiation therapy. During recovery due to a cerebro-spinal leak from the surgical site, a lumbar drain was positioned. Sudden onset of fever and increased inflammatory markers (elevated values of C-reactive protein and PCR) appeared and follow-up MR images of the spine demonstrated the presence of an intradural collection in the lumbo-sacral region (L3-L4 level), with marked restricted diffusion on DWI and mild postcontrastographic enhancement; in addition, there were signs of arachnoiditis affecting the roots of the cauda which were particularly glued together, in an antigravity position (Fig. 2). The patient initiated i.v. meropenem and fosfomycin after lumbar puncture tested positive for *Pseudomonas aeruginosa*.

He has not undergone new surgical procedures in both the cranial and spinal areas due to his poor clinical conditions.

## Discussion

Intradural spinal abscesses are extremely rare in comparison to epidural ones [3]. Most cases of intradural abscesses reported in the literature are hematogenous in origin and are more rarely associated with epidural injections and spondylodiscitis [4,5] (Table 1). This can be partially attributed to the epidural space acting as a filter. This condition is more common in older males (over the fifth decade of life), with the lumbar as the most common site of involvement [2].

**Table 1:** Review of the literature on intradural abscess.

Authors	Title	Journal	MRI findings	Intraoperative findings	Pathological findings	Etiopathogenesis
Argersinger et al. [5]	Intradural cauda equina *Candida* abscess presenting with hydrocephalus: case report	Journal of Neurosurgery: Spine	MRI of the brain: significant ventriculomegaly, transependymal flow, and abnormal enhancement at the outflow of the fourth ventricle”. MRI the spine: Diffuse leptomeningeal enhancement and an enhancing L5 intradural cauda equina mass.	Intradural location was confirmed. Marked arachnoiditis with intradural granulation tissue engulfing the caudal nerve roots. Intraoperative ultrasound revealed the L5 mass to be a loculated fluid collection.	*Candida albicans*	Immunocompromised patient with substance abuse history
Cheon et al. [3]	Pyogenic intradural abscess of lumbar spine: a case report	Korean Journal of Neurotrauma	Elongated shape, rim-enhancing cystic lesion at the ventral side of the spinal canal (L2-L4 level) causing displacement and severe compression of the dural sac. Initially thought to be epidural abscess.	Epidural space free of inflammation. A thin-walled abscess cavity at the ventral side of the intradural space.	No growth of micro-organism at the pus culture	Bacteremia occurring after operation of skin lesion located at the lower sacrum
Saigal et al. [4]	Thoracic intradural *Aspergillus* abscess formation following epidural steroid injection	American Journal of Neuroradiology	Two distinct intradural abscesses in the lower thoracic level; one of these was located intradurally at the T10-T11 level, and the other was seen dorsally at the T12–L1 level.	Intradural thick-walled cystic collection containing frank pus.	*Aspergillus fumigatus*	Epidural steroid injection performed 6 wk before presentation
Velnar and Bunc [1]	Abscess of cauda equina presenting as lumboischialgic pain: a case report	Folia Neuropathologica	Intradural 2 cm tumorous formation at L2, slightly on the left side of the spinal cord. No disc pathology. Radiologically classified as intradural neurinoma.	Pus drainage →elastic oval abscess of grey color on the left side of the conus medullaris, strongly adherent to the spinal nerves forming the cauda equina.	Sterile swabs	Lumbar puncture performed 6 months before the onset of symptoms
Kulkarni et al. [2]	Pyogenic intradural abscess	The Spine Journal	Isointense lesion at precontrast T1-weighted image, with ring enhancement after contrast medium injection.	Thick tenacious yellowish-brown fluid escape from the tense dural sac.	*Staphylococcus aureus*	Intradural pyogenic abscess secondary to chronic pyogenic spondylodiscitis
Shtaya and Hettige [6]	Disco vertebral osteomyelitis causing intradural spinal abscess with cauda equina compression	British Journal of Neurosurgery	L1-2 discitis/osteomyelitis; encapsulated, well-circumscribed, tumor-like infectious mass with restricted diffusion on DWI/ADC.	Intradural extramedullary mass	*Escherichia coli*	Discitis/osteomyelitis causing intradural extramedullary spinal abscess due to E. coli
Our study			Case 1: Intradural purulent collections in the lumbo-sacral region at L2-S2 level. Pathological hypersignal on STIR-weighted images of the left facet joint at level L2-L3. Case 2: intradural collection in the lumbo-sacral region (L3-L4 level).	Case 1: An L5-S1 decompressive laminectomy showed the epidural space free of pathological condition. Case 2: -	Case 1: No micro-organism growth neither at purulent nor at blood cultures. Case 2: Pseudomonas aeruginosa	Case 1: Most likely related to a spondylitic phenomena (involving the left facet joint at L2-L3 level). Case 2: Probable iatrogenic nature (secondary to lumbar drain positioning).

In both our cases, MRI images led us to suspect that the localization of the infectious collections was inside the intradural space. Indeed, the dural profile appeared as a continuous ipo-intense line (0.5-1 mm) delimiting the epidural fat, on the external side, from the abscess collections, on the internal side. A pial involvement was present due to the residual adhesiveness of caudal roots with antigravity position appreciable at MRI images in both cases.

In case 1, the intradural localization of the abscesses, confirmed by surgery, was probably related to spondylitis phenomena and a secondary dural breach at the left L2-L3 interfacetal joint. As described in the literature it is possible that transient bacteremia from a self-limited infection site may have been the underlying cause of interfacetal inflammation [6]. As reported on spinal epidural abscesses, *Staphylococcus aureus* is the most predominant pathogen isolated [7]. In our patient, despite abundant purulent samples being collected, a negative bacterial culture was obtained. An increase of culture-negative patients is reported in clinical practice [8] and an empirical use of vancomycin represents an appropriate therapy in these cases [8], as in our patient.

In case 2 due to the unstable clinical condition of the patient, who was bedridden, surgery was not recommended and antibiotic treatment alone was initiated. Follow-up MRI images showed a slight reduction in the size of the lumbar intradural collection.

## Conclusions

Intradural abscess is a rare and potentially dangerous condition. Despite its rarity, a high level of suspicion must alert the radiologist when dealing with a patient with intense back pain, neurological symptoms, and increased inflammatory markers. Generally, if promptly diagnosed, surgical drainage, combined with antibiotic treatment, results in a favorable outcome.

## Patient consent

I hereby confirm that I have obtained written informed consent from the patients for the publication of their cases.
